# The Acute Effect of Various Doses of Caffeine on Power Output and Velocity during the Bench Press Exercise among Athletes Habitually Using Caffeine

**DOI:** 10.3390/nu11071465

**Published:** 2019-06-27

**Authors:** Michal Wilk, Aleksandra Filip, Michal Krzysztofik, Adam Maszczyk, Adam Zajac

**Affiliations:** Institute of Sport Sciences, Jerzy Kukuczka Academy of Physical Education in Mikolowska 72a, 40-065 Katowice, Poland

**Keywords:** supplement, resistance exercise, speed, repetition

## Abstract

Background: Previously studies confirm ergogenic effects of caffeine (CAF); however there is no available scientific data regarding the influence of acute CAF intake on power output in athletes habitually consuming CAF. The main goal of this study was to assess the acute effect of 3, 6, 9 mg/kg/b.m. doses of CAF intake on power output and bench press bar velocity in athletes habitually consuming CAF. Methods: The study included 15 healthy strength-trained male athletes (age = 26.8 ± 6.2 years, body mass = 82.6 ± 9.7 kg; BMI = 24.8 ± 2.7; bench press 1RM = 122.3 ± 24.5 kg). All participants were habitual caffeine consumers (5.2 ± 1.2 mg/kg/b.m.; 426 ± 102 mg of caffeine per day). This study had a randomized, crossover, double-blind study design where each participant performed four different experimental sessions, with one week interval between each trial. In every experimental session participants performed bench press, three sets of five repetitions at 50% 1RM. The power output and bar velocity assessments under four different conditions: a placebo (PLAC), and three doses of caffeine ingestion: 3 mg/kg/b.m. (CAF-3), 6 mg/kg/b.m. (CAF-6) and 9 mg/kg/b.m. (CAF-9). Results: The statistical significance was set at *p* < 0.05. The repeated measures ANOVA between PLAC and CAF-3; CAF-6; CAF-9 revealed no statistically significant differences in power output and velocity of the bar during the bench press exercise. A large effect size (ES) in mean power-output was found between PLAC and CAF-9 in Sets 1 and 2. A large ES in peak power-output was found between PLAC and CAF-6 in Set 2, and between PLAC and CAF-9 in Sets 1 and 2. A large ES in peak velocity was found between PLAC and CAF-9 in Sets 1–3. Conclusion: The results of the present study indicate that acute doses of CAF before exercise does not have a significant effect on power output and bar velocity in a group of habitual caffeine users.

## 1. Introduction

Resistance training is a significant component of conditioning programs in competitive sports. The ability to generate high values of power output is one of the most significant factors determining success in numerous sport disciplines [1]. Power output can be described by the relationship between the force generated by the muscles and movement velocity [2]. Particular attention in studies concerning the development of power and high speed of movement has been directed at exercise volume with specific intensity of effort [3,4]. In addition to training, nutrition and supplementation also have a significant effect on adaptation and post-exercise responses [5,6,7,8,9].

Caffeine (CAF) is among the most often used and widely studied supplements in competitive sports. Mechanisms responsible for ergogenic effects of CAF are linked to the impact on various tissues, organs and systems of the human body. In the central nervous system (CNS), CAF acts through interactions with adenosine receptors that influence the release of noradrenaline, dopamine, acetylcholine and serotonin [10,11,12,13] and consequently, increase muscle tension [14]. Increased muscle activation can lead to a greater energy demand during exercise, thus leading to a faster depletion of energy substrates in muscle cells [15]. 

Numerous studies have examined the acute performance-enhancing effects of CAF intake on human physical fitness and exercise performance [16,17,18,19,20,21,22,23,24]. The most frequently consumed dose of caffeine ranges from 3 to 9 mg/kg body mass (b.m.), ingested in the form of capsules 30 to 90 minutes before exercise. However, the optimal dose may differ based on exercise choice, volume, intensity, and the type of muscle contraction [23,25,26,27,28]. Additionally, participants characteristics, such as gender, age and training experience can affect both, power output and the ergogenic effects of CAF intake. Although ergogenic effect of CAF is well-established in many aspects, much controversy remains about the effectiveness of different doses of caffeine on power output of the upper limbs.

Previous studies showed positive acute effects of 3 mg/kg b.m. of CAF on resistance exercise performance and power output, suggesting that this dose has significant ergogenic properties [25,29,30]. However a dose of 3 mg/kg b.m. is sufficient to increase movement velocity at loads of 25–50%1RM, whereas a higher caffeine dose (9 mg/kg b.m.) is necessary when submaximal loads (90%1RM), are used despite the appearance of adverse side effects [25]. Grgic and Mikulic [24] showed an increase in power output during a medicine ball throw following CAF intake (6 mg/kg b.m.). Pallarés et al. [25] also showed significantly increased movement velocity and power output at loads of 25–50%1RM after different doses of CAF ingestion (3, 6, 9 mg/kg b.m.), however, at the load of 75% 1RM, a CAF dose of 3 mg/kg b.m. did not improve power output in the bench press exercise. On the contrary, the study of Wilk et al. [20] did not show changes in concentric power output and bar velocity during the bench press to concentric muscle failure, following the intake of 5 mg/kg/b.m. of CAF compared to a placebo. 

Furthermore, one should emphasize that most of the previous studies on CAF intake and the level of power output concerned participants with low daily CAF intake. In competitive athletes the use of CAF before resistance exercise is particularly common. As a result, research suggesting 75–90% of athletes consume CAF before or during training sessions and competitive events [31,32]. According to Svenningsson et al. [33], Fredholm et al. [34] habitual CAF intake modifies physiological responses to acute ingestion by the up-regulation of adenosine receptors. Furthermore, constant exposure to CAF could impact metabolic pathways by inducing cytochrome P450 1A2 and increased induction speed of that enzyme which may alter the rate of CAF metabolism. However based on the available evidence, it does not seem that habitual caffeine ingestion reduces the ergogenic benefits of acute CAF supplementation [35,36,37]. Evans et al. [38] suggested that non-habitual CAF (<40 mg/day) users experience a greater magnitude of the ergogenic effect compared with CAF habitual users (>130 mg/day). However, Gonçalves et al. [36] indicate that habitual CAF intake (low = 58; moderate = 143; high = 351 mg/day) did not influence exercise performance, suggesting that CAF habituation has no detrimental impact on CAF ergogenesis. Likewise Dodd et al. [35] also did not show any differences in time to exhaustion after acute doses of CAF (placebo; 3 mg/kg/b.m.; 5 mg/kg/b.m.), between a non-habitual user group (<25 mg/day) and habitual CAF consumers (>300 mg/day). The basic source of variability of results among scientists investigating habitual caffeine use, is the division of subjects into low, moderate, and high habitual caffeine consumption groups. Some studies have defined high caffeine use as >100 mg/day [39], while others have defined it as >300 mg/day [35] or even >750 mg/day [40]. Inconsistency in these caffeine intake reference values makes the interpretation and cross-comparison of results difficult. Furthermore, daily doses of CAF intake are reported in values of mg/day [36,38,41], which lacks precision and may be very misleading when athletes with different body mass are considered. This lack of consistency in caffeine use levels has been noted before, and yet, to our knowledge, no one has proposed reference values for caffeine use. 

Since there is no available scientific data regarding the influence of acute CAF intake on power output in athletes habitually consuming CAF, with a precisely determined intake of CAF in relation to body mass (mg/day/kg/b.m.) the main goal of this study was to assess the acute effect of various doses of CAF on power output and bar velocity in athletes habitually consuming CAF (4–6 mg/day/kg/b.m.). 

## 2. Materials and Methods

### 2.1. Study Participants

Fifteen (*n* = 15) healthy strength-trained male basketball and handball athletes participated in the study after completing an ethical consent form (age = 26.8 ± 6.2 years, body mass = 82.6 ± 9.7 kg, BMI = 24.8 ± 2.7, bench press 1RM = 122.3 ± 24.5 kg; data presented as mean ± standard deviation [SD]) with a minimum 3 years of strength training experience (4.2 ± 1.23 years). All participants were habitual caffeine consumers (5.2 ± 1.2 mg/kg/b.m., 426 ± 102 mg of caffeine per day). The inclusion criteria were as follows: (a) free from neuromuscular and musculoskeletal disorders, (b) the participants were able to perform the bench press exercise with a load of at least 120% of their body mass [42], (c) habitual caffeine intake in the range of 4–6 mg/kg/b.m., ~300–500 mg of caffeine per day. The study protocol was approved by the Bioethics Committee for Scientific Research, at the Academy of Physical Education in Katowice, Poland (10/2018) according to the ethical standards of the Declaration of Helsinki, 1983.

### 2.2. Habitual Caffeine Intake Measurement

Habitual caffeine intake was assessed by a specific Food Frequency Questionnaire (FFQ) adapted from a previously questionnaires [43] under the supervision of a qualified nutritionist. The questionnaire was employed to assess the habitual consumption of dietary products rich in caffeine. Portions, in household measures, were used to assess the amount of food consumed according to the following frequency of consumption: a) more than three times a day, b) two to three times a day, c) once a day, d) five to six times a week, e) two to four times per week, f) once a week, g) three times per month, h) rarely or never. The list was composed of dietary products with high caffeine content including different types of coffees, teas, energy drinks, cocoa’s products, popular beverages, medications and caffeine supplements. Previously published information and nutritional tables were used for database construction [17,44,45]. Based on the answers in FFQ, a qualified nutritionist estimated the habitual caffeine intake.

### 2.3. Experimental Designed

This study used a randomized, crossover, double-blind design where each participant performed a familiarization session with a 1RM test on one day, and four different experimental sessions with one week interval between each trial. The randomization was conducted by a member of the research team that was not directly involved in the data collection. Participants underwent the power output and bar velocity assessments under four different conditions: a placebo (PLAC), and three doses of caffeine ingestion: 3 mg/kg/b.m. (CAF-3), 6 mg/kg/b.m. (CAF-6) and 9 mg/kg/b.m. (CAF-9). CAF or a PLAC were administered orally 60 minutes before each exercise protocol to allow peak blood caffeine concentration and at least 2 hours after the last meal to maintain the same time of absorption. CAF was provided in the form of standard capsules containing 300 mg of CAF, as well as those specifically prepared for the research, containing 100, 50 and 5 mg doses of CAF. The PLAC was provided in identical capsules as CAF (all-purpose flour). All CAF and PLAC capsules were manufactured by Olimp Laboratories. Subjects refrained from physical activity other than that required by the experimental trials and withdrew from alcohol, tobacco and other drugs and supplements during the study. The participants were instructed to maintain their usual hydration, dietary habits including habitual caffeine intake during the entire experiment and keep track of their calorie intake using the “Myfitness pal” software [46] every 24 hours before the testing procedure. There were no differences between individually calorie intake between particular sessions. The subjects were also asked to refrain from heavy exercise for 48 hours and to refrain from caffeine intake 12 hours before each trial. All testing was performed in the Strength and Power Laboratory at the Jerzy Kukuczka Academy of Physical Education in Katowice.

### 2.4. Familiarization Session and One Repetition Maximum Test

A familiarization session preceded the one repetition maximum testing. The participants arrived at the laboratory at the same time of day as the upcoming experimental sessions (in the morning between 9:00 and 10:00 am) and cycled on an ergometer for 5 minutes at an intensity that resulted in a heart rate of around 130 bpm, followed by a general upper body warm-up. Next, the participants performed 15, 10, and 5 repetitions of the bench press exercise using 20%, 40%, and 60% of their estimated 1RM with a 2/0/X/0 tempo of movement [42]. The sequence of digits describing the tempo of movement (2/0/X/0) represents a 2 second eccentric phase, in which 0 represents a pause during the transition phase, X represents maximum possible tempo of movement during the concentric phase, and the last digit represents no pause at the end of movement. The participants then executed single repetitions with a 5 minutes rest interval between successful trials. The load for each subsequent attempt was increased by 2.5 kg, and the process was repeated until failure. Hand placement on the barbell was individually selected with a grip width on the barbell of 150% individual bi-acromial distance (BAD). BAD was determined by palpating and marking the acromion with a marker, and then measuring the distance between these points with a standard anthropometric tape. The positioning of the hands was recorded to ensure consistent hand placement during all testing sessions. No bench press suits, weightlifting belts, or other supportive garments were permitted. Three spotters were present during all attempts to ensure safety and technical proficiency. 

### 2.5. Experimental Protocol

Four testing sessions were used for the experimental trials. All testing took place between 9.00 and 11.00 a.m. to avoid circadian variation. The general warm-up for the experimental sessions was identical to the one used for the familiarization session. After the warm-up, participants started the main examinations and performed three set of the bench press with 5 repetitions in each set at 50%1RM. The concentric phase of movement was performed at maximal possible velocity, while the eccentric phase with a 2 second duration (2/0/X/0). All repetitions were performed without bouncing the barbell off the chest, without intentionally pausing at the transition between the eccentric and concentric phases, and without raising the lower back off the bench. The time between each session of the experiment was 7 days. During the experimental trials the participants were encouraged to perform at maximal engagement according to the recommendations by Brown and Weir [47]. A linear position transducer system “Tendo Power Analyzer” (Tendo Sport Machines, Trencin, Slovakia) was used for the evaluation of bar velocity. The Tendo Power Analyzer is a reliable system for measuring movement velocity and power output [48,49]. The system consists of a velocity sensor connected to the load by a kevlar cable which, through an interface, instantly transmits the vertical velocity of the bar to a specific software installed in the computer (Tendo Power Analyzer Software 5.0). The system measures upward vertical average and peak velocity of the movement. Using a set external load, the system calculates average peak power and peak velocity in the concentric phase of the movement. The measurement was made independently for each repetition and automatically converted into the values of peak power output (PP), mean power output (MP), peak velocity (PV), mean velocity (MV). All participants completed the described testing protocol. 

### 2.6. Side Effects

Immediately and after 24 hours following each testing procedures participants answered a side effects questionnaire, included nine items on a yes/no scale of caffeine ingestion [25,50,51,52].

#### Statistical Analysis

The Shapiro–Wilk, Levene and Mauchly´s tests were used in order to verify the normality, homogeneity and sphericity of the sample data variances. Verification of differences between the PLAC and CAF-3, CAF-6, CAF-9 was performed using ANOVA with repeated measures. Effect sizes (Cohen’s d) were reported where appropriate. Parametric effect sizes (ES), were defined as large for d > 0.8, as moderate between 0.8 and 0.5, and as small for <0.5 [52,53], and was calculated at 95% confidence intervals. The statistical significance was set at *p* < 0.05. All statistical analyses were performed using Statistica 9.1 and Microsoft Office, and were presented as means with standard deviations.

## 3. Results

The repeated measures ANOVA between PLAC and CAF-3; CAF-6; CAF-9 revealed no statistically significant differences in MP (Table 1), PP (Table 2), MV (Table 3) as well PV (Table 4). No significant differences in PP, MP, PV, MV between PLAC and CAF-3; CAF-6; CAF-9 were observed for Sets 1–3. However, a large effect size (ES) in MP was found between PLAC and CAF-9 in Set 1 and 2 (Table 1). Similarly, the large ES in PP was found between PLAC and CAF-6 in Set 2, and between PLAC and CAF-9 in Set 1 and 2 (Table 2). Additionally, the large ES in PV was found between PLAC and CAF-9 in Sets 1–3 (Table 4).

### Caffeine Side Effects

Table 5 details the nine different side effects assessed immediately and 24 hours later. Immediately after the PLAC trial, subjects reported a very low frequency of side effects (0%–13%; QUEST + 0 hours). The CAF-3 treatments produced very similar side effects (0%–20%; QUEST + 0 hours), compared with the PLAC trial. The CAF-6 treatments produced greater value of side effects (0%–47%; QUEST + 0 hours). The greatest value of side effect was recorded for perception of performance and increased vigor (40%–47%; QUEST + 0 hours). Finally, the CAF-9 trial produced a drastic increase in the reported frequency of side effects (0%–87%; QUEST + 0 hours) (Table 5). 

The following morning of each experimental trial (QUEST + 24 hours), very few participants (0%–7%) reported that PLAC treatment produced residual side effects. The CAF-3 trial produced very similar side effects to PLAC (0%–13%; QUEST + 24 hours). The CAF-6 trial showed greater frequency of side effects, with increased urine output and headaches in comparison with the PLAC and CAF-3 conditions, although with a frequency lower than 33% of the subjects. Finally, CAF-9 increased the frequency of all adverse side effects, with a frequency of appearance from 0 to 73%. In the group ingesting the highest dose of CAF, 67%–73% of participants reported tachycardia, anxiety or nervousness, gastrointestinal problems, and 53% had increased urine output (Table 5).

## 4. Discussion

The main finding of the study was that acute CAF intake has no significant effect on PP, MP, PV, MV in habitual users of caffeine. Significant changes in PP, MP, PV, MV were not registered after CAF intake with doses of 3, 6 or 9 mg/kg/b.m. compared to PLAC. Despite the fact that the results of our study are inconsistent with previous findings [24,25,26] it should be emphasized that this is the first scientific study which considers the acute effect of different doses of CAF intake on power output and bar velocity changes during the bench press exercise in habitual CAF users.

Previous research showed that acute CAF intake increase power output [24,25]. However, most of the studies concerned participants with low daily CAF intake. Actually, there are only a few studies analyzing acute effects of CAF intake in habitual users; however, the results are not conclusive and mostly refer to aerobic endurance exercises [35,36,41]. To the best of our knowledge, only one study analyzed power output changes of the upper limbs after different doses of acute CAF intake in group of habitual users [26]. The study of Sabol et al. [26] showed an increase in medicine ball throwing distance in subjects ingesting 6 mg/kg/b.m. of CAF compared to a PLAC. However the differences were non-significant between the intake of PLAC and 2 mg/kg/b.m. as well between PLAC and 4 mg/kg/b.m. It is worth noting that the study of Sabol et al. [26] did not show significant differences in responses to acute CAF ingestion between the groups of low and moderate-to-high habitual CAF users. On the contrary, the result of our study did not show any significant changes in power output and bar velocity after the intake of CAF with a dose of 3, 6 or 9 mg compared to the PLAC. However, it must be indicated that in the study of Sabol et al. [26] only six participants were classified as those with moderate-to-high habitual CAF intake. Furthermore, this group had a very wide range of daily CAF intake (CAF = 358 ± 210 mg/day; range = 135 to 642 mg/day), which limits the reliability of results. 

Our study is the first of its kind with a homogeneous research group (*n* = 15) with study participants consuming CAF in the range of 4 to 6 mg/kg/b.m. (~300–500 mg/day). Differences related to the daily CAF intakes limit the possibility to compare our results to those of Sabol et al. [26]. Other previous studies with habitual CAF users, also used different criteria for daily CAF intake. Gonçalves et al. [36] applied the following reference values for daily CAF intake: low = 58 ± 29 mg/day; moderate = 143 ± 25 mg/day; high = 351 ± 139 mg/day. On the other hand Sabol et al. [26] considered high consumers as subjects ingesting >100 mg/day of CAF, however in the study of Dodd et al. [35] habitual CAF users were defined as subjects that consumed > 300 mg/day. Differences in daily CAF consumption as well lack of reference values to body mass limits the possibility of comparing previous research results. Furthermore, our study is the first in which the daily intake of CAF was determined in relation to body mass.

The physiological effects of acute CAF intake in habitual caffeine consumers is relatively unstudied. Previous research has suggested that high habitual caffeine intake may reduce the ergogenic effects of acute CAF supplementation on exercise performance [54], what was confirmed in our study. However Pickering and Kiely [54] suggested that reductions after acute CAF intake in habitual users can be modified by using pre-trial doses, substantially greater than habitual intake. While this idea has been perpetuated in the scientific literature, the results of our study confirmed this statement. The result of our study did not show significant changes in PP, MP, PV, MV after acute CAF intake compared to PLAC, even when greater pre-trial doses (CAF-9) were used compared to habitual daily intake (4–6 mg/kg/b.m./day). 

The results of our study showed that the habitual caffeine intake limits physiological responses to acute CAF doses, in agreement with Svenningsson et al. [33] and Fredholm et al. [34]. Caffeine is an adenosine receptor antagonist, and when ingested, it binds to adenosine receptors [55]. In animal models, studies reported that chronic caffeine intake increases adenosine receptor concentration and this increase attenuates caffeine’s effects [33]. In humans, given that the ergogenic effects of caffeine are strongly linked to its effects on adenosine receptors, it has been suggested that habitual caffeine users may experience smaller enhancement in performance following acute CAF intake as compared to non-users [41]. However, exercise itself may alter the sensitivity of adenosine receptors and lower the threshold concentration such that a smaller dose provided during or at the beginning of exercise may be equally or more effective than similar or larger doses provided 1 hour prior to exercise [56,57]. Likewise, smaller doses provided during warm-up exercise immediately prior to performance testing [58], or immediately prior to and during exercise [59] can be ergogenic and may be as effective as a single larger dose ingested 1 hour prior to exercise [60]. However there is no data available about changes in sensitivity of adenosine receptors in physically active, habitual CAF users.

Despite the fact that our study did not show significant changes in power output and bar velocity after acute CAF intake compared to PLAC, it should be noted that there was a large ES in MP, PP, PV between CAF-9 and PLAC which, indicates an acute ergogenic effect. While such changes in power output and bar velocity might be considered as small in statistical terms, this difference may be of great significance in training of elite athletes as well as in scientific research. It is known that plasma levels of caffeine needed to induce tissue changes are significantly higher than those required to affect adenosine receptors in the brain and peripheral nervous system [61,62], which may explain the occurrence of side effects using acute intake of CAF-6 and CAF-9 (Table 5), despite the lack of significant changes in power output and bar velocity. Therefore it can be concluded that acute intake of CAF in habitual users is to some extent ergogenic.

The variety of methodological approaches and results obtained make meaningful conclusions and recommendations for athletes difficult. Furthermore, it is hard to isolate the direct effects of CAF from systematic effects due to the number of potential mechanisms evoked from its wide distribution within the body. Although there is some controversy in regard to caffeine dose–response relationship, it is suggested that caffeine intake increases adrenaline release, evokes greater Ca^2+^ release from the sarcoplasmic reticulum, improves the function of the Na^+^/K^+^ pump, reduces pain perception and increases plasma fatty acid concentration [63,64]. The present study has several limitations which should be addressed. There were no genetic assessments related to CAF intolerance in the tested athletes. However, according to studies of Cornelis et al. [65] genetic variation in the A2A receptor, the main target of caffeine action in the CNS, is associated with caffeine consumption. Probability of having the ADORA2A 1083TT genotype associated with caffeine-induced anxiety [66] decreases as the caffeine intake increases in a population, and subjects with that genotype are more likely to limit their caffeine intake. People who were homozygous for the 1083T allele experienced greater anxiety after consuming 150 mg of caffeine [66]. Furthermore, before the start of our experiment no study participant reported any side effects after consumption of caffeine within the previous six months.

## 5. Conclusions

The results of the present study indicate that acute doses of CAF before exercise does not have a significant effect on power output and bar velocity of the bar during the bench press exercise in a group of habitual caffeine users. No significant changes in the above mentioned variables were observed at each of the three doses of CAF administered (3, 6, 9 mg/kg/b.m.). However the results of our study refer only to power output and bar velocity of the upper limbs during the bench press exercise with an external load of 50%1RM. These results therefore may not translate to other forms, volumes, or intensities of exercise.

## Figures and Tables

**Table 1:** Results of mean power output in three successive sets of the bench press exercise in the group that ingested different doses of caffeine, and the placebo group.

	Mean Power [W]										
	Placebo (95% CI)	Caffeine 3 mg (95% CI)	*p*	ES	Caffeine 6 mg (95% CI)	*p*	ES	Caffeine 9 mg (95% CI)	*p*	ES	*F*
Set 1	445 ± 98 (403; 508)	453 ± 96 (402; 504)	0.99	0.51	462 ± 92 (413; 511)	0.99	0.55	464 ± 98 (411; 516)	0.99	0.93	0.04
Set 2	456 ± 92 (407; 505)	465 ± 97 (413; 516)	0.99	0.48	474 ± 98 (422; 526)	0.94	0.47	457 ± 77 (416; 498)	0.99	0.82	0.13
Set 3	463 ± 93 (413; 513)	456 ± 93 (407; 506)	0.99	0.42	469 ± 99 (416; 522)	0.99	0.36	473 ± 102 (418; 528)	0.99	0.58	0.08

Notes: mean ± standard deviation [SD]; CI: confidence interval.

**Table 2:** Results of peak power output in three successive sets of the bench press exercise in the group that ingested different doses of caffeine, and the placebo group.

	Peak Power [W]										
	Placebo (95% CI)	Caffeine 3 mg (95% CI)	*p*	ES	Caffeine 6 mg (95% CI)	*p*	ES	Caffeine 9 mg (95% CI)	*p*	ES	*F*
Set 1	831 ± 171 (740; 922)	874 ± 202 (767; 982)	0.90	0.59	843 ± 167 (754; 932)	0.99	0.61	848 ± 169 (752; 945)	0.99	0.8	0.16
Set 2	819 ± 172 (727; 911)	874 ± 198 (768; 979)	0.81	0.41	879 ± 175 (785; 973)	0.76	0.93	821 ± 136 (752; 899)	0.99	0.81	0.54
Set 3	858 ± 181 (728; 921)	846 ± 176 (752; 941)	0.98	0.46	871 ± 173 (779; 963)	0.88	0.62	869 ± 172 (773; 968)	0.99	0.72	0.22

Notes: mean ± standard deviation [SD]; CI: confidence interval.

**Table 3:** Results of mean velocity in three successive sets of the bench press exercise in the group that ingested different doses of caffeine, and the placebo group.

	Mean Velocity [m/s]										
	Placebo (95% CI)	Caffeine 3 mg (95% CI)	*p*	ES	Caffeine 6 mg (95% CI)	*p*	ES	Caffeine 9 mg (95% CI)	*p*	ES	*F*
Set 1	0.94 ± 0.08 (0.90; 0.99)	0.90 ± 0.07 (0.86; 0.94)	0.35	0.53	0.93 ± 0.06 (0.89; 0.96)	0.90	0.51	0.91 ± 0.05 (0.88; 0.94)	0.62	0.73	1.01
Set 2	0.94 ± 0.08 (0.90; 0.99)	0.93 ± 0.09 (0.88; 0.98)	0.98	0.50	0.95 ± 0.07 (0.91; 0.99)	0.99	0.51	0.90 ± 0.06 (0.87; 0.94)	0.53	0.77	0.96
Set 3	0.95 ± 0.08 (0.91; 1.00)	0.92 ± 0.09 (0.87; 0.97)	0.69	0.44	0.94 ± 0.08 (0.90; 0.99)	0.99	0.52	0.93 ± 0.05 (0.90; 0.96)	0.88	0.61	0.47

Notes: mean ± standard deviation [SD]; CI: confidence interval.

**Table 4:** Results of peak velocity in three successive sets of the bench press exercise in the group that ingested different doses of caffeine, and the placebo group.

	Peak Velocity [m/s]										
	Placebo (95% CI)	Caffeine 3 mg (95% CI)	*p*	ES	Caffeine 6 mg (95% CI)	*p*	ES	Caffeine 9 mg (95% CI)	*p*	ES	*F*
Set 1	1.42 ± 0.16 (1.33; 1.51)	1.42 ± 0.16 (1.33; 1.50)	0.99	0.41	1.38 ± 0.12 (1.31; 1.44)	0.81	0.79	1.38 ± 0.11 (1.32; 1.45)	0.89	0.81	0.37
Set 2	1.40 ± 0.16 (1.31; 1.49)	1.43 ± 0.16 (1.34; 1.52)	0.96	0.64	1.44 ± 0.16 (1.35; 1.52)	0.93	0.71	1.36 ± 0.14 (1.28; 1.44)	0.88	0.92	0.68
Set 3	1.42 ± 0.16 (1.34; 1.51)	1.43 ± 0.20 (1.32; 1.54)	0.99	0.51	1.43 ± 0.14 (1.35; 1.51)	0.99	0.55	1.41 ± 0.13 (1.34; 1.48)	0.99	0.82	0.05

Notes: mean ± standard deviation [SD]; CI: confidence interval.

**Table 5:** Side effects reported by participants immediately after the testing protocol (QUEST + 0 hours) and 24 hours later (QUEST + 24 hours).

Side Effects	Doses of CAF Intake During Testing Protocol							
	PLAC		CAF 3 mg/b.m.		CAF 6 mg/b.m.		CAF 9 mg/b.m.	
	+0 h	+24 h	+0 h	+24 h	+0 h	+24 h	+0 h	+24 h
Muscle soreness	0	0	0	0	0	0	0	0
Increased urine output	1 (7%)	1 (7%)	3 (20%)	2 (13%)	6 (40%)	5 (33%)	10 (67%)	8 (53%)
Tachycardia and heart palpitations	2 (13%)	1 (7%)	3 (20%)	2 (13%)	6 (40%)	3 (20%)	12 (80%)	11 (73%)
Anxiety or nervousness	1 (7%)	1 (7%)	2 (13%)	7 (7%)	3 (20%)	2 (13%)	10 (67%)	3 (20%)
Headache	2 (13%)	1 (7%)	3 (20%)	1 (7%)	2 (13%)	4 (26%)	3 (20%)	6 (40%)
Gastrointestinal problems	0	1 (7%)	2 (13%)	1 (7%)	4 (26%)	2 (13%)	6 (40%)	11 (73%)
Perception of performance improvement	2 (13%)	-	3 (20%)	-	6 (40%)	-	13 (87%)	-
Increased vigor/activeness	2 (13%)	1 (7%)	2 (13%)	1 (7%)	7 (47%)	2 (13%)	13 (87%)	6 (40%)
Insomnia	-	0	-	0	-	2 (13%)	-	4 (26%)

Data are presented as number of person (n) as well the percentage of prevalence (%).
